# Progesterone Beyond Pituitary Suppression in Assisted Reproduction: Peripheral Signalling, Gamete Biology, Endometrial Competence and Precision Therapeutic Use

**DOI:** 10.3390/ijms27208944

**Published:** 2026-10-09

**Authors:** Aron Łukaszuk, Jakub Łukaszuk, Izabela Wocławek-Potocka, Grzegorz Jakiel, Krzysztof Łukaszuk

**Affiliations:** 1 INVICTA Fertility Clinic, 80-850 Gdańsk, Poland; aron.lukaszuk@invicta.pl; 2 Centre of Postgraduate Medical Education, 01-004 Warsaw, Poland; grzegorz.jakiel1@o2.pl; 3 INVICTA Research and Development Center, 81-740 Sopot, Poland; jakub.lukaszuk@invicta.pl; 4 Embryo Biology Team, InLife Institute of Animal Reproduction and Food Research, Polish Academy of Sciences, 10-683 Olsztyn, Poland; i.woclawek-potocka@pan.olsztyn.pl; 5 Department of Reproductive Medicine and Gynecological Endocrinology, Medical University of Gdańsk, 80-210 Gdańsk, Poland

**Keywords:** progesterone receptor, PGRMC1, PAQR, CatSper, oocyte competence, decidualization, luteal phase support, frozen embryo transfer, pharmacokinetics, precision medicine

## Abstract

Progesterone is indispensable to assisted reproduction, yet the phrase “progesterone effect” collapses non-equivalent biological processes. Classical progesterone receptors (PGR-A and PGR-B), progestin and adipoQ receptor-family proteins, progesterone receptor membrane components 1 and 2 (PGRMC1/2), and an unresolved proximal sensor upstream of sperm CatSper operate over different timescales and with unequal evidence for direct ligand binding. In the ovary, PGRMC-associated complexes regulate granulosa-cell survival, mitosis and oocyte competence, although much causal evidence is non-human. In sperm, progesterone-evoked CatSper activation is robust, whereas the influential ABHD2 hydrolase model was challenged by human data in 2026. In endometrium, receptivity emerges from temporally ordered PGR-dependent epithelial–stromal, immune, metabolic and vascular programmes; “progesterone resistance” is therefore better treated as a family of molecular states than as a single diagnosis. Oral, vaginal, subcutaneous and intramuscular regimens generate different systemic and uterine exposures, and serum progesterone is an exposure marker rather than a direct assay of tissue response. We integrate these levels into an exposure–target engagement–tissue state–outcome framework. This framework explains why universal serum thresholds and unselected rescue strategies remain uncertain and defines the experiments and trials needed for mechanism-informed luteal support and frozen embryo transfer.

## 1. Introduction

Progesterone is used so routinely in assisted reproductive technology (ART) that its mechanistic complexity is easily hidden by a single drug name and a single serum concentration. In fresh in vitro fertilization (IVF), ovarian stimulation and oocyte-maturation triggers disrupt the endogenous luteal phase, making exogenous support a standard component of care. In programmed frozen embryo transfer (FET), treatment replaces an absent corpus luteum as the principal source of progesterone. In ovulatory FET, exogenous progesterone may instead supplement an endogenous, pulsatile source. These are not interchangeable physiological states, and neither are the available formulations or routes of administration [1,2,3].

The same problem exists at the molecular level. The term “progesterone receptor” can refer to the classical nuclear receptor encoded by *PGR*, to members of the progestin and adipoQ receptor (PAQR) family historically called membrane progestin receptors, or to PGRMC1 and PGRMC2. These proteins differ in structure, subcellular location and strength of evidence for direct progesterone binding. Rapid progesterone responses may also be produced by membrane-associated classical PGR or by signalling complexes whose proximal ligand sensor remains uncertain. Equating expression, pharmacological perturbation and direct target engagement has generated an appealing but overconfident receptor taxonomy [4,5,6,7].

Three examples make this distinction clinically consequential. First, progesterone rapidly increases intracellular calcium in human sperm through CatSper, but the identity and mechanism of the upstream progesterone sensor remain unsettled after evidence that ABHD2 catalytic activity is not required [8,9,10,11]. Second, PGRMC1/2 perturbation changes granulosa-cell survival, cell-cycle progression and oocyte maturation, yet these multifunctional haem-associated proteins cannot automatically be treated as conventional ligand-activated progesterone receptors [7,12,13,14]. Third, serum progesterone around embryo transfer is associated with outcome in several settings, but route-dependent uterine delivery, assay variability, sampling time and tissue competence prevent a circulating threshold from being a universal surrogate for endometrial response [1,15].

This review develops a peripheral and tissue-centred account of progesterone in ART. We examine sperm, ovary, oocyte, endometrium and luteal-support pharmacology, while deliberately excluding hypothalamic–pituitary suppression, the preovulatory luteinizing hormone surge and progestin protocols used during ovarian stimulation. Those topics require a separate neuroendocrine evidence framework. Here, the central question is different: how should molecular target identity, tissue exposure and cellular state be connected to clinically meaningful outcomes without converting association into mechanism?

Our principal proposal is an exposure–target engagement–tissue state–outcome model. “Exposure” includes route, dose, formulation, adherence, sampling and corpus-luteum context. “Target engagement” requires evidence that a ligand reaches and functionally engages a defined molecular system. “Tissue state” encompasses the spatially and temporally coordinated response of sperm, ovarian cells or endometrial compartments. “Outcome” includes fertilization, implantation, miscarriage and live birth. The framework is intentionally restrictive: a change at one level cannot be assumed to prove the next. It is intended to make progesterone research more falsifiable and clinical decisions more transparent.

## 2. Review Approach and Evidence Interpretation

We conducted a targeted narrative search of the PubMed/MEDLINE database through 15 May, and then again through 21 August 2026, supplementing it with backward and forward citation tracking in mechanistic reviews, randomized controlled trials, systematic reviews, and key primary studies.

Search concepts combined progesterone or progestogens with PGR isoforms, PAQR/membrane progestin receptors, PGRMC1/2, CatSper/ABHD2, granulosa cells, oocyte competence, decidualization, endometrial receptivity or resistance, luteal phase support, FET, pharmacokinetics, serum monitoring and rescue supplementation. The complete reproducible queries are provided in Supplementary Table S1. We preferentially used human studies for clinical inference, but retained non-human experiments when they supplied causal or spatial information not obtainable in humans. Full texts available by 30 August 2026 were inspected for the priority mechanistic, pharmacokinetic and clinical claims; the verification record and remaining access gaps are reported in the Supplementary Materials.

Evidence was interpreted by claim rather than by article. For receptor identity, the most persuasive sequence is direct ligand binding with appropriate controls, structural or biophysical confirmation, genetic perturbation, rescue and a temporally coherent downstream phenotype. Immunoreactivity or a response to a nominally selective agonist is insufficient on its own. For tissue mechanisms, we distinguished correlation from perturbation and tracked species, cell model, differentiation state, ligand concentration and exposure time. For clinical claims, randomized evidence and live birth were prioritized over biochemical pregnancy; systematic reviews were interpreted in light of regimen, route and cycle-context heterogeneity. Observational rescue studies were treated as estimates of a treatment strategy only when their design addressed immortal-time bias, confounding by indication and the use of post-baseline serum measurements.

This is not a systematic review or a meta-analysis. The search was designed to map a mechanistically heterogeneous field and to evaluate the inferential bridge between molecular and clinical evidence. Accordingly, absence from this narrative synthesis should not be interpreted as evidence of absence. Conversely, inclusion of a molecular association does not imply clinical utility.

## 3. A Receptor and Signalling Taxonomy Based on What Has Actually Been Demonstrated

### 3.1. Classical PGR: Isoforms, Chromatin and Rapid Signalling

The classical progesterone receptor is a ligand-activated transcription factor expressed principally as PGR-A and PGR-B. The isoforms share DNA- and ligand-binding domains but differ in their N termini and can exert overlapping, opposing or context-dependent transcriptional effects. Their relative abundance, post-translational modification, cofactor availability and chromatin accessibility help determine the cellular response. In human endometrial stromal cells, progesterone-dependent decidualization reorganizes the PGR transcriptome and cistrome rather than simply switching on a fixed panel of genes [5,16,17].

Classical PGR should not be equated exclusively with slow genomic signalling. A fraction of the receptor can participate in rapid kinase and membrane-proximal signalling through protein interactions. Reported extranuclear actions include coupling to Src-family kinase, MAPK/ERK and PI3K/AKT signalling, with the magnitude and direction of the response depending on PGR isoform, cell type and experimental system. These rapid signals may also modify later transcriptional responses, but they must be distinguished experimentally from PAQR- and PGRMC-associated pathways [4,5]. However, rapid timing alone does not identify the receptor. Demonstrating a seconds-to-minutes response establishes kinetics, not target identity. Studies should combine isoform-resolved loss of function, ligand-binding controls and rescue with compartment-specific constructs before assigning a rapid response to membrane-associated PGR.

The clinical implication is that the same circulating progesterone concentration can produce different transcriptional programmes in tissues with different PGR-A:PGR-B ratios, co-regulators or epigenetic states. It also explains why a single biopsy marker is unlikely to summarize the endometrium’s integrated response to progesterone.

### 3.2. PAQR-Family Proteins: Plausible Rapid Signalling, Uneven Receptor Evidence

PAQR5, PAQR7 and PAQR8 are commonly referred to as membrane progestin receptors γ, α and β, respectively. Experimental systems have linked these proteins to rapid changes in second messengers, cyclic nucleotide signalling, kinase activity and cellular function. PAQR7 has received particular attention in female reproductive tissues [6,18]. Nevertheless, the literature is difficult to compare because expression systems, antibodies, ligands and readouts differ, and because endogenous receptor abundance is often low or poorly localized.

The correct conclusion is not that PAQR-mediated progesterone signalling is disproven, but that the evidential strength varies by protein and tissue. A functional response after overexpression, or blockade by a compound marketed as selective, does not establish physiological ligand binding in an endogenous complex. Future studies need quantitative binding, genetic rescue at endogenous expression levels, orthogonal target-engagement assays and explicit exclusion of classical PGR and other steroid-sensitive pathways.

### 3.3. PGRMC1 and PGRMC2: Multifunctional Nodes, Not Automatically Conventional Receptors

PGRMC1 and PGRMC2 are membrane-associated, haem-binding proteins with roles in cytochrome P450 regulation, trafficking, metabolism, cell survival and division. They can occur in multiprotein complexes and show tissue- and cell-cycle-dependent localization. Progesterone-associated phenotypes have been reported after PGRMC1/2 perturbation in ovarian and endometrial models, but the name “progesterone receptor membrane component” is not itself proof of a classical receptor mechanism [7,19,20].

This distinction matters because genetic removal of a multifunctional scaffold can alter steroid synthesis, membrane trafficking, mitochondrial state or mitosis and thereby change a progesterone-sensitive phenotype without being the direct ligand-binding event. A rigorous PGRMC claim should specify whether the evidence demonstrates direct binding, complex membership, necessity, sufficiency or downstream modulation. Throughout this review, we therefore use “PGRMC-associated” unless target engagement has been independently demonstrated.

### 3.4. Natural Progesterone and Synthetic Progestogens Are Not Mechanistically Interchangeable

Micronized progesterone, dydrogesterone, medroxyprogesterone acetate and other progestogens differ in oral bioavailability, metabolite profile, affinity for PGR isoforms, off-target activity at androgen, glucocorticoid or mineralocorticoid receptors, and potential effects on sperm ion channels. Evidence obtained with one ligand cannot be transferred automatically to another. This is especially important when molecular studies use supraphysiological concentrations or compounds assumed to be receptor-selective. In ART, clinical efficacy of an oral progestogen may coexist with a different systemic and tissue pharmacology from natural vaginal or injectable progesterone [21,22,23].

**Figure 1:**
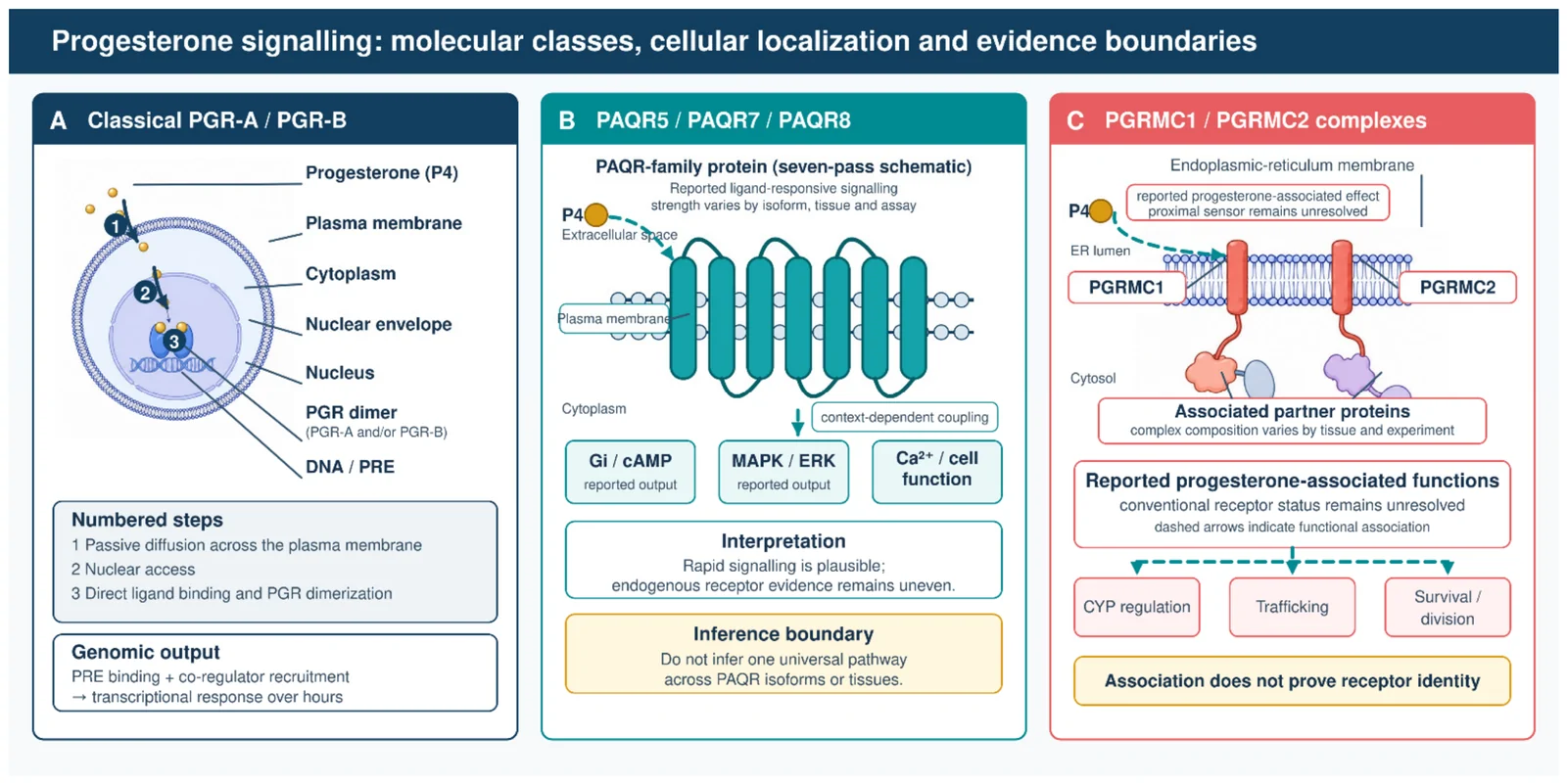
Molecular classes, cellular localization and evidence boundaries of progesterone signalling. (**A**) Simplified canonical genomic signalling through classical progesterone receptor (PGR) isoforms. Progesterone (P4) diffuses across the plasma membrane, gains access to the nucleus, binds PGR-A and/or PGR-B, and promotes receptor dimerization. PGR dimers bind progesterone-response elements (PREs) and recruit co-regulators to modify transcription over hours. Rapid extranuclear actions of classical PGR are not shown. (**B**) PAQR5, PAQR7 and PAQR8 are represented as seven-pass PAQR-family membrane proteins. Ligand-responsive signalling and reported Gi/cAMP, MAPK/ERK and Ca^2+^/cell-function outputs are isoform-, tissue-, expression-system- and assay-dependent. These connections should therefore not be interpreted as one universal PAQR pathway. (**C**) PGRMC1 and PGRMC2 are single-pass, endoplasmic-reticulum-associated haem-binding proteins functioning in context-dependent multiprotein complexes. Reported associations with CYP regulation, trafficking and cell survival or division do not establish that PGRMC1/2 are conventional progesterone receptors or identify the proximal progesterone sensor. Solid numbered arrows in panel A indicate canonical steps; dashed arrows in panels B and C indicate reported functional associations or unresolved proximal sensing.

Figure 1 and Table 1 organize these systems by molecular claim and evidential burden. The taxonomy is not merely semantic: it determines what can be inferred when a drug changes a phenotype.

## 4. Human Sperm: A Robust Progesterone Response with an Unresolved Proximal Sensor

### 4.1. CatSper Is the Central Effector of the Rapid Calcium Response

Human sperm encounter progesterone in the female reproductive tract, including in the cumulus–oocyte complex. Nanomolar-to-micromolar progesterone exposure can evoke a rapid intracellular calcium rise and alter flagellar behaviour. Two independent studies in 2011 established the CatSper-pathway dependence of this response in human sperm [8,9]. Strünker et al. detected the onset of the calcium signal within the 36 ms resolution of their system and reported half-maximal responses in the low-nanomolar range, with values dependent on the endpoint and capacitation state [9]. Lishko et al. showed marked potentiation of human CatSper current by 500 nM progesterone, whereas the homologous mouse current did not show this response [8]. These experiments establish rapid, species-selective CatSper activation; they do not identify direct progesterone binding to the pore-forming channel or settle the identity of the proximal sensor. Subsequent work characterized human CatSper as a polymodal chemosensor integrating voltage, intracellular pH and reproductive-tract signals [24,25].

The phenotype is mechanistically and clinically compelling. Men with disrupted CatSper function can have impaired progesterone-induced calcium entry and compromised fertilizing capacity despite sperm concentrations or routine motility measures that do not fully disclose the defect [26,27]. Yet, the existence of a robust channel response does not identify the molecule that binds progesterone.

### 4.2. The ABHD2 Model Was Influential but Is No Longer a Complete Explanation

In 2016, an elegant model proposed that progesterone activates the α/β hydrolase-domain protein ABHD2, which hydrolyses the endocannabinoid 2-arachidonoylglycerol and relieves tonic CatSper inhibition [10]. The pathway connected steroid sensing, lipid metabolism and ion-channel gating, and it rapidly became the default explanation for non-genomic progesterone action in human sperm.

New human evidence requires the model to be revised. Edwards and colleagues reported in 2026 that ABHD2 activity was not required for the non-genomic action of progesterone on human sperm; their results did not support ABHD2 hydrolase activity as the necessary link to CatSper and did not establish direct progesterone binding as the operative event [11]. This does not erase the possibility that ABHD2 modulates sperm lipid signalling or CatSper under some conditions. It does mean that “progesterone binds ABHD2, ABHD2 hydrolyses 2-AG, CatSper opens” should no longer be presented as a settled linear pathway.

The revised model has a firm core and an open front end. CatSper-dependent calcium influx is the firm effector. The proximal sensor, its molecular stoichiometry, and the contribution of ABHD2 catalytic versus non-catalytic functions remain open. Candidate mechanisms must also accommodate donor heterogeneity, capacitation state, pH and exposure history.

### 4.3. Translational Opportunities and Limits

**Figure 2:**
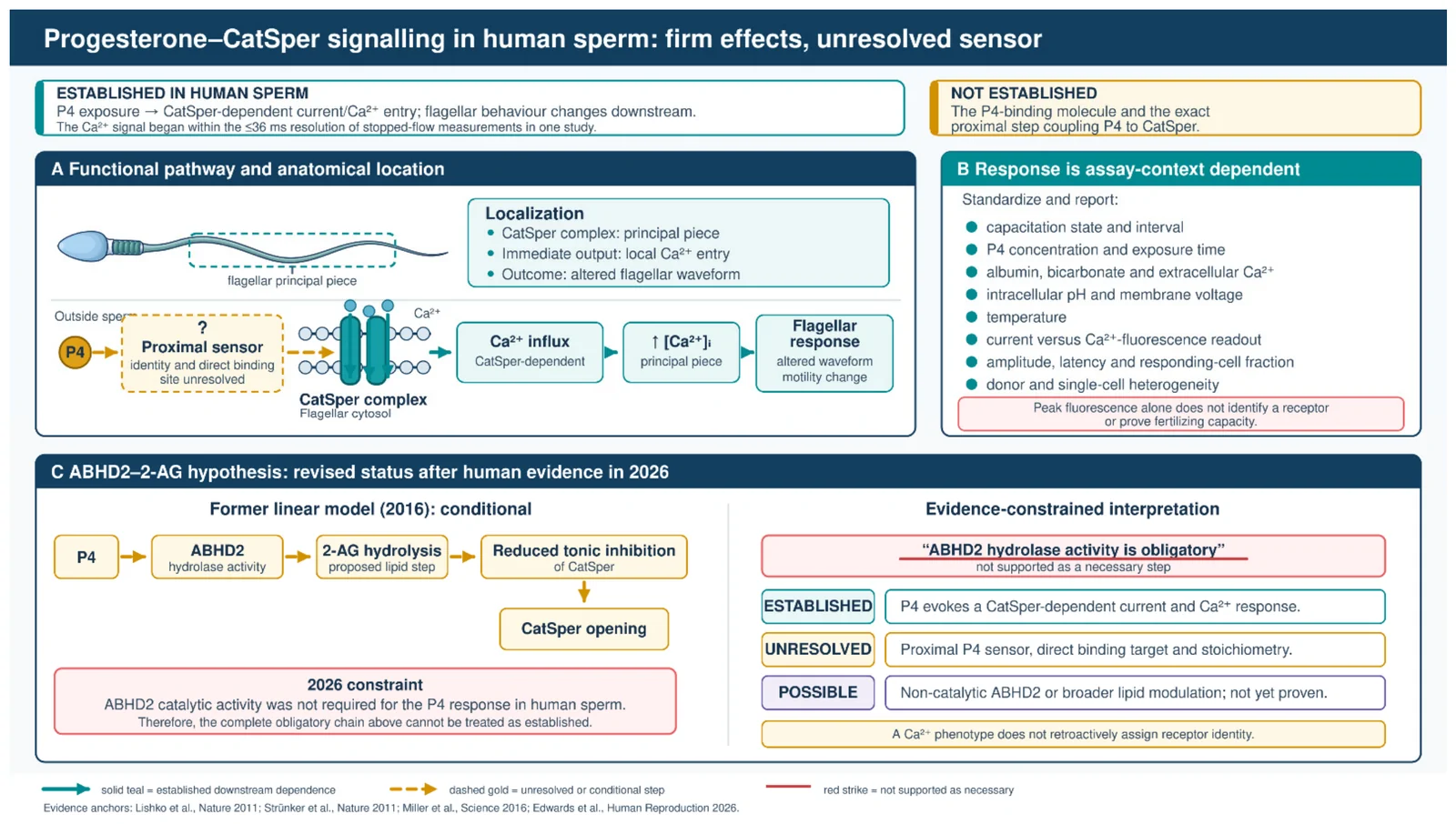
Evidence-constrained model of progesterone-evoked CatSper signalling in human sperm. (**A**) Progesterone (P4) evokes a rapid CatSper-dependent current and Ca2+ influx in the flagellar principal piece, increasing local intracellular Ca2+ and altering flagellar behaviour. This downstream functional link is well supported, whereas the proximal P4-binding molecule and the exact coupling step remain unresolved. (**B**) The measured response depends on capacitation state and interval, P4 concentration and exposure time, medium composition, intracellular pH, membrane voltage, temperature, readout, and donor- and single-cell heterogeneity. A fluorescence peak alone neither identifies a receptor nor proves fertilizing capacity. (**C**) The influential 2016 model proposed that alpha/beta hydrolase domain-containing protein 2 (ABHD2) hydrolyses 2-arachidonoylglycerol (2-AG), thereby relieving tonic CatSper inhibition. Human evidence published in 2026 showed that ABHD2 catalytic activity was not required for the non-genomic P4 response; therefore, the complete P4–ABHD2–2-AG–CatSper chain is shown as conditional rather than obligatory. Non-catalytic ABHD2 or broader lipid-modulatory roles remain possible but unproven. Solid teal arrows denote established downstream dependence; dashed gold arrows denote unresolved or conditional steps; a red strike denotes a step not supported as necessary. Supporting references: Lishko et al. [8], Strünker et al. [9], Miller et al. [10], and Edwards et al. [11].

Progesterone-evoked calcium assays or CatSper-responsive motility phenotyping might reveal functional sperm defects missed by conventional semen analysis (Figure 2). Before use as diagnostics, however, assays require standardized progesterone concentration, albumin and bicarbonate conditions, temperature, capacitation interval, calcium indicator, response metric and reference population. The relevant endpoint is not simply a peak fluorescence value; amplitude, latency, responding-cell fraction, oscillation and flagellar localization may encode different defects.

Synthetic progestogens add another translational uncertainty. Steroids and related compounds can modulate CatSper in ligand-specific ways [23]. A clinical luteal-support regimen is unlikely to expose ejaculated sperm in vivo in the same way as an acute laboratory challenge, but pharmacological findings reinforce the rule that “progestogenic” activity at nuclear PGR does not predict CatSper activity.

The next decisive experiments should combine rapid ligand photoaffinity labelling or chemoproteomics in native human sperm with CatSper electrophysiology, ABHD2 catalytic-dead and null perturbations in tractable models, and rescue by precisely localized constructs. Human sperm are transcriptionally silent and experimentally difficult, but that limitation makes direct target-engagement evidence more—not less—important.

## 5. Ovary and Oocyte: PGRMC-Associated Control of Survival, Division and Competence

### 5.1. Granulosa-Cell Survival

Follicular development depends on the survival and coordinated differentiation of granulosa and cumulus cells around the oocyte. Progesterone-associated anti-apoptotic effects and PGRMC1-dependent mechanisms have been described in ovarian somatic-cell models. Early work localized PGRMC1 in the immature rat ovary and linked it to progesterone-mediated inhibition of granulosa-cell apoptosis [28]. In human granulosa/luteal cells, progesterone promoted survival through a PGRMC1-dependent mechanism [12]. The bovine study by Okuda and colleagues provides a complementary but indirect pharmacological observation: blocking endogenous progesterone action with 10−4 M onapristone in cultured mid-luteal cells increased TUNEL positivity from 3.1% to 37.1%, increased Fas/caspase-3 signalling and enhanced cell death, including with Fas ligand [29]. Because progesterone was not added as a rescue intervention and the responsible receptor was not identified, this experiment supports tonic progesterone-dependent survival in a progesterone-rich compartment but does not demonstrate a PGRMC-specific mechanism.

These studies support a survival function, but several inferential steps remain. Cell-culture progesterone concentrations may exceed free intrafollicular exposure; luteinized granulosa cells obtained after stimulation differ from preovulatory mural granulosa cells; and PGRMC1 loss can alter metabolism or membrane trafficking independently of direct progesterone sensing. It is therefore more accurate to conclude that PGRMC1 is necessary for some progesterone-associated survival phenotypes than to call every such phenotype proof that PGRMC1 is the ligand-binding receptor.

### 5.2. Cell-Cycle Progression, Spindle Organization and Chromosome Segregation

In spontaneously immortalized rat granulosa cells, PGRMC1 and PGRMC2 interact, redistribute during the cell cycle and suppress inappropriate entry into division [13]. In a subsequent study, conditional granulosa-cell Pgrmc1 deletion in mice was combined with mechanistic experiments in spontaneously immortalized granulosa cells; the results linked PGRMC1/2-dependent control of mitosis and survival to NF-κB/p65 localization and activity [14]. PGRMC1 localization to the mitotic spindle and midbody, together with perturbation phenotypes during late mitosis, suggests that these proteins participate in chromosome segregation and cytokinesis rather than acting only as membrane steroid sensors [30].

The functional consequences extend beyond proliferation. Abnormal granulosa-cell division can change follicle architecture, paracrine support, steroidogenesis and the composition of follicular fluid. However, an observed association between PGRMC abundance and embryo outcome in an IVF cohort would still be vulnerable to confounding by follicle size, gonadotropin exposure, trigger-to-retrieval interval and degree of luteinization. Mechanistic studies should therefore measure cell-cycle state and steroidogenic differentiation together.

### 5.3. Oocyte Meiotic State and Competence

PGRMC-associated functions have been reported in the oocyte itself. PGRMC1 participates in bovine oocyte meiosis and has been localized in relation to the spindle [30]. Progesterone–PGRMC1 signalling suppressed meiotic prophase I progression in mouse oocytes in one experimental model [31]. Reviews of bovine oocyte development further connect PGRMC1 with competence, although they emphasize the incompleteness of the pathway [32]. Genetic studies in zebrafish and mice also support reproductive roles for PGRMC proteins, including effects on oocyte maturation, luteal vascularization and maintenance of the primordial-follicle pool [33,34,35].

Species and developmental timing are decisive. Human oocytes retrieved for IVF have been exposed to controlled ovarian stimulation, a maturation trigger and a peri-retrieval follicular environment that differs from most animal models. An effect on maintenance of meiotic arrest in an immature follicle cannot be extrapolated directly to post-trigger oocyte competence. Likewise, a spindle-localized protein may be essential to meiosis but not be acting as a progesterone receptor during that event.

### 5.4. Follicular Fluid as a Systems-Level Readout

Follicular fluid integrates secretions from granulosa and theca cells, plasma transudate, inflammatory mediators, lipids and metabolites. Proteomic and peptidomic studies demonstrate substantial within-person and between-follicle complexity and illustrate why a single progesterone value cannot define oocyte competence [36,37]. Recent analyses from members of the present author group identified protein and pathway differences associated with embryo development and with polycystic ovary syndrome (PCOS), reinforcing a systems-level view of the follicular microenvironment [38,39]. These studies were not designed to establish a progesterone receptor mechanism, and they should not be cited as doing so. Their relevance lies in defining the biological background against which progesterone and PGRMC-associated effects operate.

Prostaglandins provide one plausible bridge among steroid signalling, inflammation and oocyte maturation. Prostaglandin E2 altered bovine oocyte maturation in vitro in work involving Wocławek-Potocka and Łukaszuk [40]. The study supports local lipid-mediator crosstalk but also exemplifies the translational boundary: a controlled bovine maturation system cannot establish a human IVF treatment effect.

### 5.5. A Human Translation Agenda

**Figure 3:**
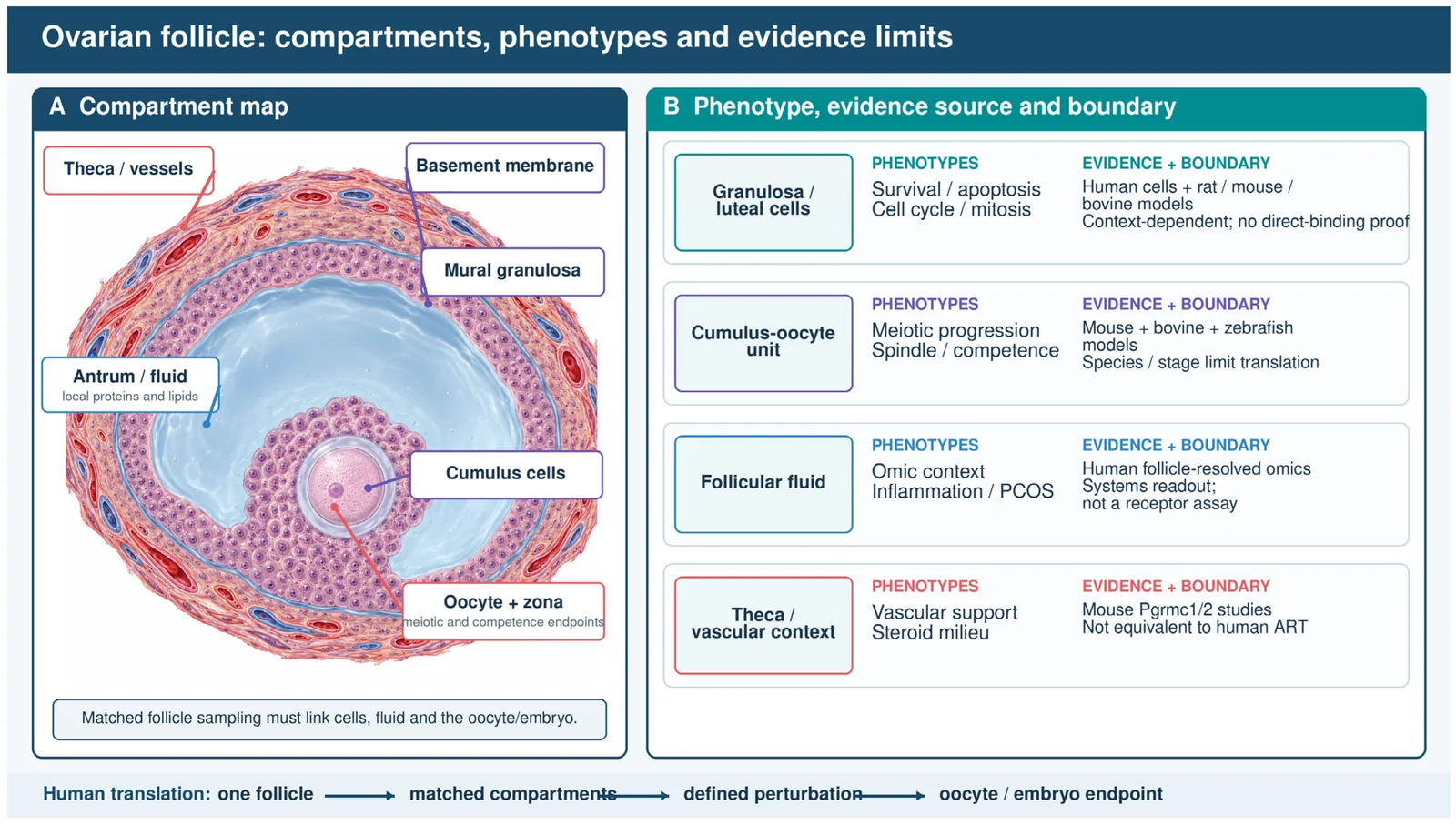
Compartment-resolved progesterone and PGRMC-associated biology in the ovarian follicle. (**A**) The compartment map identifies the theca and vessels, basement membrane, mural granulosa, antrum and follicular fluid, cumulus cells, and the oocyte with zona pellucida. Leader lines terminate on the named structure. (**B**) Reported phenotypes are separated from their strongest evidence source and principal translational limitation. Granulosa/luteal survival and cell-cycle phenotypes are supported by human-cell perturbation and rat, mouse and bovine models; oocyte meiotic and spindle phenotypes remain predominantly non-human. Human follicular-fluid omics provides systems-level context but is not a receptor assay. PGRMC1/2 localization or loss of function may establish necessity for a phenotype, but does not by itself establish conventional receptor identity or direct P4 binding to the operative complex. Human translation requires follicle-resolved linkage of mural granulosa, cumulus, follicular fluid and the corresponding oocyte or embryo.

The most useful human studies would combine follicle-resolved sampling with spatial and functional measurements. Mural granulosa, cumulus cells, follicular fluid and the corresponding oocyte or embryo should be linked without pooling across follicles. PGRMC1/2 abundance and localization should be measured alongside PGR isoforms, steroid concentrations, lipid mediators, cell-cycle state and mitochondrial function (Figure 3 and Table 2). If clinical outcomes are studied, fertilization, blastocyst development and euploidy should be separated from implantation, because the latter introduces endometrial and transfer-related causes.

Organoid and microfluidic follicle systems may help test causality while preserving cell–cell communication. Perturbations should include genetic depletion and rescue, with variants designed to separate haem binding, complex formation and putative steroid-interaction functions. Until such data exist, PGRMC1/2 are best regarded as promising integrators of ovarian cell state rather than validated clinical biomarkers.

## 6. Endometrium: From Progesterone Exposure to a Coordinated Tissue State

### 6.1. Decidualization Is a Temporal Network, Not a Binary Switch

After estrogen priming, progesterone transforms the endometrium from a proliferative tissue into a secretory, receptive and subsequently decidual state. This process requires coordinated responses in luminal and glandular epithelium, stromal fibroblasts, immune populations and vasculature. In human stromal cells, decidualization reorganizes PGR binding and transcription at a genome-wide scale [16]. PGR-A and PGR-B make non-identical contributions, and their functional balance changes with differentiation state [17]. The endpoint cannot therefore be reduced to the expression of one receptor or one canonical marker.

FOXO1 is a central node in the decidual network and is required for PGR occupancy at regulatory elements that include the transcriptional regulator IRF4 [41]. GATA2 physically and functionally interacts with PGR during stromal decidualization [42]. Progesterone-regulated insulin receptor substrate 2 links hormone signalling to metabolic competence [43]. These examples illustrate a broader principle: PGR action is conditioned by lineage-specific transcription factors and by the energetic and inflammatory state of the cell.

### 6.2. Epithelial–Stromal and Immune Communication

Endometrial competence is an emergent property of communication among compartments. Human endometrial epithelial organoid secretions can alter stromal decidualization, showing that epithelial polarity and basolateral signalling matter [44]. Progesterone also reshapes chemokines, prostaglandins, cytokines and immune-cell behaviour. A review by Sieg summarized inflammation-related molecules at the maternal–fetal interface and in pathologically altered endometrium [45]. Its appropriate role here is to support the inflammatory and prostaglandin context, not to substitute for direct evidence of a progesterone mechanism.

The implantation window is therefore not a fixed clock reading that can be inferred from progesterone exposure alone. It reflects embryo stage, epithelial adhesiveness, glandular secretion, stromal differentiation, immune tolerance and vascular adaptation. Molecular receptivity tests capture selected components of this state and can be affected by sampling time and tissue composition. Biopsy itself also perturbs the tissue, making retrospective molecular classification easier than prospective treatment selection.

### 6.3. PGRMC Proteins in Decidualization

PGRMC1 and PGRMC2 are expressed in endometrium and have been implicated in uterine physiology and disease [19,46]. Recent work reported a progesterone-associated PGRMC1 rise-to-decline trajectory during decidualization, highlighting that the direction and timing of change may be more informative than a single abundance measurement [47]. For PGRMC2, Medina-Laver et al. analyzed 77 endometrial biopsies from 66 healthy, fertile oocyte donors and observed cycle-dependent expression, with a mid-secretory increase followed by a late-secretory decline [48].

In primary human endometrial stromal cells, decidualization induced with cAMP plus medroxyprogesterone acetate increased PGRMC2 mRNA and protein. PGRMC2 knockdown altered 4687 transcripts and 28 proteins and impaired the capacity of decidualized stromal cells to support JEG-3 trophoblast-spheroid expansion [48]. The convergent transcriptomic, proteomic and functional observations strengthen the case for a PGRMC2-associated decidualization programme, but the omics and spheroid experiments were small (RNA-seq n = 4, SWATH proteomics n = 5 and outgrowth n = 6), used a synthetic progestin rather than natural progesterone, and lacked the epithelial, immune and vascular compartments of intact endometrium. They neither demonstrate direct progesterone binding to PGRMC2 nor prove an in vivo implantation effect.

These findings fit a dynamic-complex model: a protein may be required early for signalling or trafficking but need to decline for terminal differentiation. They also caution against classifying low or high expression as uniformly favourable. A static immunohistochemical score cannot reveal whether the tissue followed the correct trajectory.

### 6.4. “Progesterone Resistance” Is a Family of Molecular States

Progesterone resistance is used in endometriosis, adenomyosis, PCOS, endometrial polyps and recurrent implantation failure, often to describe poor response despite apparently adequate exposure. The umbrella term is useful clinically but mechanistically underspecified. Proposed lesions include altered PGR-A:PGR-B balance or methylation, inflammatory kinase signalling, deficient co-regulators, aberrant estrogen metabolism, SIRT1-related programmes, metabolic dysfunction and disrupted epithelial–stromal communication [49,50,51,52,53].

PCOS provides a clear example of heterogeneity. Endometrial progesterone resistance has been described in association with hyperandrogenism and metabolic dysfunction [54]. Experimental work indicates that androgen excess can impair human stromal decidualization through AMPK/SIRT1/PDK4-related metabolic changes [55]. This does not imply that all patients with PCOS share one endometrial lesion or that a higher progesterone dose corrects it. Exposure failure and response failure must be distinguished.

Recurrent implantation failure is similarly heterogeneous. EHD1-associated impairment of decidualization has been linked to SENP1 and PGR signalling [56], but a molecular pathway identified in selected cells should not be converted directly into a universal clinical test. A useful classification should identify the affected level: inadequate exposure, altered receptor abundance or isoform balance, cofactor/chromatin failure, inflammatory interference, metabolic insufficiency or incorrect timing.

### 6.5. Measuring Tissue Competence

**Figure 4:**
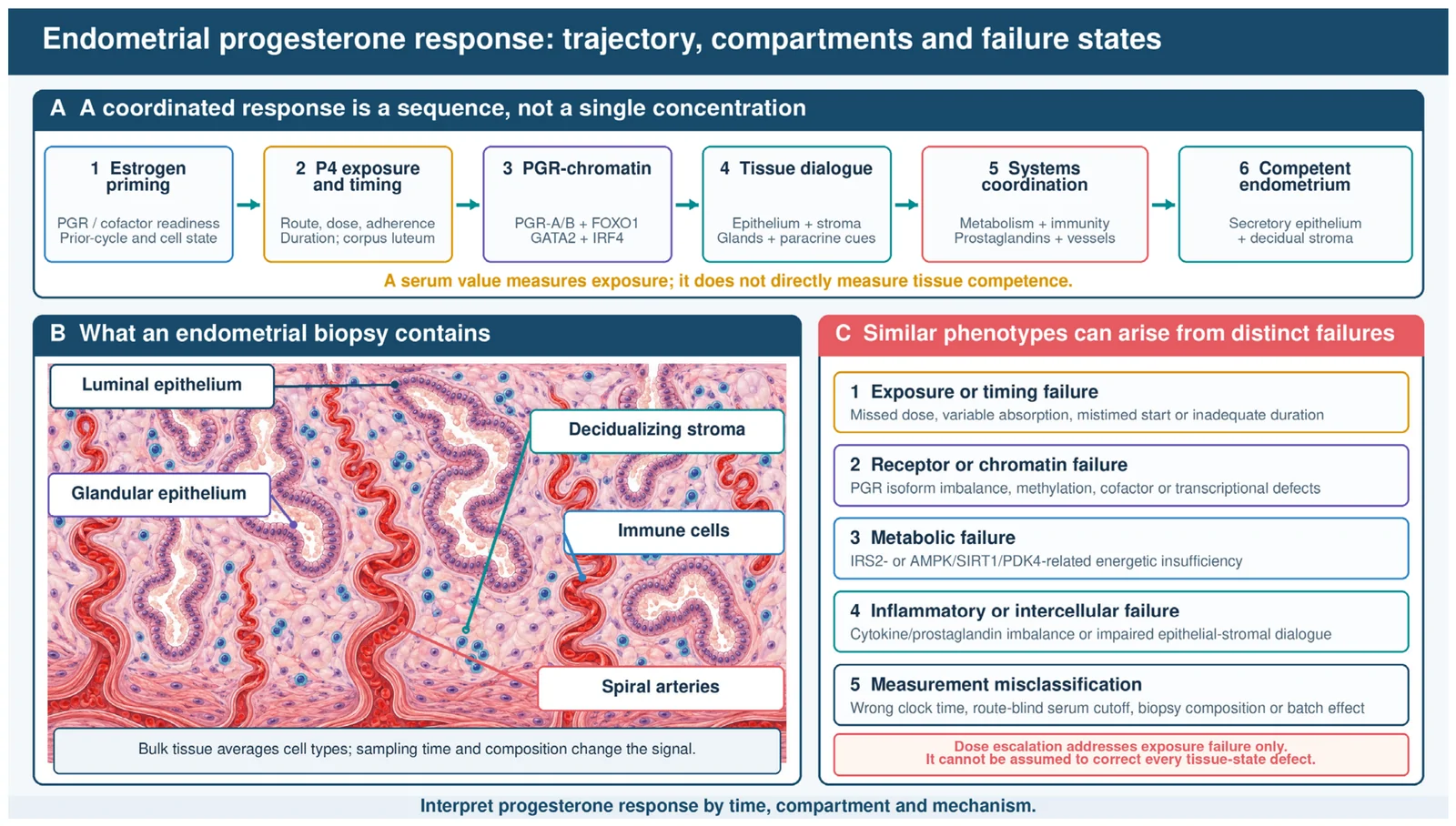
Temporal and compartmental architecture of the endometrial progesterone response. (**A**) A competent endometrial state requires estrogen priming, correctly timed and route-specific P4 exposure, classical PGR-dependent chromatin activity, epithelial-glandular-stromal dialogue, and metabolic, immune, prostaglandin and vascular coordination. Serum progesterone measures exposure rather than tissue competence. (**B**) The labelled tissue section distinguishes luminal epithelium, glandular epithelium, decidualizing stroma, immune cells and spiral arteries. Bulk measurements therefore depend on sampling time and compartment composition. (**C**) Similar poor decidualization or implantation phenotypes may arise from exposure/timing failure, receptor/chromatin failure, metabolic insufficiency, inflammatory/intercellular dysfunction or measurement misclassification. Increasing P4 dose can address exposure failure, but cannot be assumed to correct every state labelled “progesterone resistance”.

An endometrial biomarker intended for treatment selection should meet four conditions. It should be reproducible across laboratories; add information beyond route, dose, adherence and serum exposure; predict a clinically relevant outcome in an external cohort; and identify an intervention that improves that outcome when assigned prospectively (Figure 4 and Table 3). Most current markers do not yet meet all four.

Single-cell and spatial transcriptomic approaches can identify cell-type-specific defects obscured in bulk tissue. Organoids and assembloids can test epithelial–stromal causality, but they lack parts of the immune and vascular environment. The most credible translational programme will connect spatial tissue state to an intervention in a randomized biomarker-strategy trial, rather than merely correlating a molecular signature with implantation.

## 7. Clinical Pharmacology: Route, Cycle Context and the Meaning of a Serum Concentration

### 7.1. The Route Creates the Exposure Profile

Progesterone can be delivered vaginally, intramuscularly, subcutaneously or orally as a progestogen with suitable bioavailability. Preferential uterine exposure after vaginal administration is commonly described by the historical “first uterine pass” concept [57,58]. Direct tissue support includes a small, unequal comparative study in 20 functionally agonadal women: 15 received vaginal progesterone 200 mg every 6 h and five received intramuscular progesterone 50 mg twice daily; vaginal treatment produced substantially higher endometrial tissue concentrations despite lower serum concentrations [59]. This establishes route-dependent serum–tissue dissociation under the studied regimens, not a universal quantitative law or a fixed tissue-to-serum ratio [57,58,59,60]. Intramuscular progesterone produces sustained systemic concentrations but adds pain, local reactions and injection burden. Subcutaneous formulations reduce needle length and may improve acceptability while still producing systemic exposure. Oral micronized progesterone undergoes extensive first-pass metabolism and is not equivalent to orally active dydrogesterone.

**Figure 5:**
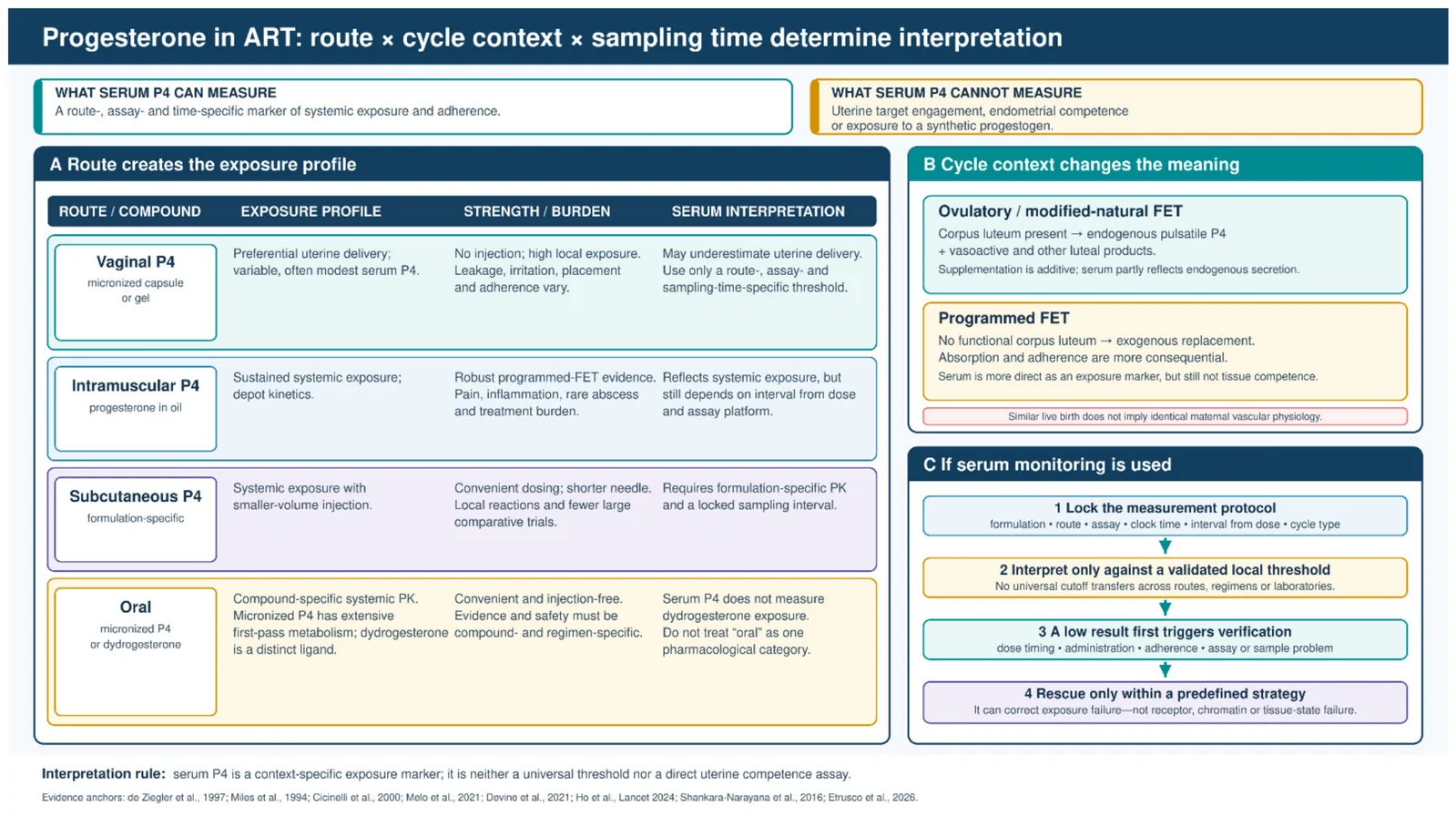
Route-, cycle-, and time-dependent interpretation of progesterone exposure in assisted reproductive technology (ART). (**A**) Vaginal, intramuscular, subcutaneous, and oral regimens produce non-equivalent systemic and uterine exposure profiles. Vaginal administration may preferentially deliver P4 to the uterus despite modest serum concentrations; intramuscular and subcutaneous regimens produce systemic exposure with route-specific kinetics and burdens. Oral micronized P4 and dydrogesterone are pharmacologically distinct, and serum P4 does not quantify dydrogesterone exposure. (**B**) Natural or modified-natural frozen embryo transfer (FET) includes endogenous, pulsatile P4 and other corpus-luteum products, whereas programmed FET relies on exogenous replacement. Similar live-birth rates do not establish identical maternal vascular physiology. (**C**) A serum P4 result is interpretable only with formulation, route, dose, adherence, assay, cycle type, clock time, and interval from the preceding dose. A low result should first trigger verification of timing, administration, adherence, and analytic factors. Protocolized rescue may correct exposure failure; it is not a treatment for receptor, chromatin, metabolic, inflammatory, or other tissue-state failure. No universal cutoff can be transferred across routes, regimens, assays, and laboratories. Supporting references: Melo et al. [15], de Ziegler et al. [57], Bulletti et al. [58], Miles et al. [59], Cicinelli et al. [60], Devine et al. [66], Ho et al. [68], Shankara-Narayana et al. [77], Stavridis et al. [78] and Etrusco et al. [84].

These profiles undermine the assumption that the route with the highest serum value necessarily produces the best uterine target engagement [Figure 5 and Table 4]. Conversely, preferential uterine delivery does not guarantee uniform endometrial exposure among patients. Vaginal placement, dissolution, mucosal factors, adherence and uterine anatomy may contribute to variability. Serum and tissue measurements answer different questions.

### 7.2. Fresh IVF: Progesterone Is Effective, but “Best” Is Regimen-Dependent

Controlled ovarian stimulation disrupts luteal physiology, and progesterone support improves ongoing pregnancy or live birth compared with placebo or no treatment, although much of the foundational randomized evidence is old and methodologically heterogeneous [2]. Contemporary reviews continue to identify uncertainty about optimal timing, dose, route and duration [1,61]. A 2024 network meta-analysis included 76 randomized trials and 26,536 participants across 22 interventions, but network complexity, historical protocols and moderate confidence limit simple ranking of every regimen [62].

Oral dydrogesterone has been compared with micronized vaginal progesterone in phase III trials and meta-analysis. LOTUS I and LOTUS II supported non-inferior efficacy for ongoing pregnancy in fresh IVF under the studied regimens [63,64]. An individual-participant-data meta-analysis reported similar safety and at least comparable pregnancy and live-birth outcomes, but interpretation should account for trial sponsorship, heterogeneity in vaginal comparators and the fact that dydrogesterone is a distinct ligand rather than merely an oral route for natural progesterone [65]. 

### 7.3. Programmed FET: Replacement Is Not the Same as Supplementation

In programmed FET, there is no functional corpus luteum unless ovulation occurs unexpectedly. Exogenous progesterone must therefore create and maintain the luteal endocrine environment. This makes regimen failure more consequential than in an ovulatory cycle with endogenous secretion.

A three-arm randomized trial assigned 1125 women undergoing programmed FET of a good-quality, non-biopsied vitrified blastocyst; 1060 completed transfer [66]. The regimens were daily intramuscular progesterone 50 mg, vaginal micronized progesterone 200 mg twice daily, or the same vaginal regimen plus intramuscular progesterone 50 mg every third day. Live birth per transfer was 27%, 44% and 46%, respectively; approximately half of pregnancies in the vaginal-only group ended in miscarriage, and that arm was stopped after an interim analysis. Using a 10-percentage-point non-inferiority margin, the combination was non-inferior to daily intramuscular administration. The result is formulation-, dose-, schedule- and population-specific: it does not establish inferiority of every vaginal regimen, does not address euploid-only transfer, and should not be generalized to natural-cycle FET.

Smaller randomized comparisons illustrate the efficacy–tolerability trade-off. A 2024 three-arm trial reported more biochemical pregnancies with intramuscular than vaginal or subcutaneous administration, but no statistically significant clinical-pregnancy difference; injection-site adverse effects and lower satisfaction favoured non-intramuscular routes [67]. Formulation, dose, embryo population and endpoint differences prevent a simple route league table.

### 7.4. Ovulatory FET and the Corpus-Luteum Context

Natural and modified-natural FET preserve a corpus luteum, although trigger and luteal-support regimens vary. A 2024 open-label trial randomized 1428 ovulatory women equally to start with natural, modified-natural or artificial endometrial preparation [68]. Live birth after one completed FET within the assigned strategy was 37%, 33% and 34%, respectively; relative risks versus artificial preparation were 1.07 (95% CI 0.87–1.33) for natural and 0.98 (0.79–1.22) for modified-natural preparation. However, 99 first cycles were cancelled in each ovulatory group versus none in the artificial group, and women with a cancelled first cycle could switch to artificial preparation for a subsequent FET. The estimand is therefore a preparation strategy leading to one completed transfer, not pure per-started-cycle biological efficacy. The single-centre setting, 35% double-embryo-transfer rate, power based on a 10-percentage-point difference and limited safety power constrain generalization. The trial supports multiple feasible strategies, not endocrine or safety equivalence.

Randomized trials of progesterone supplementation in natural or modified-natural FET have not been uniform. Some reported improved live birth with vaginal progesterone, whereas others did not show benefit under their specific timing and trigger conditions [69,70,71,72]. The biological reason is plausible: endogenous luteal sufficiency, embryo stage and treatment timing differ. Trials should report ovulation confirmation, corpus-luteum number, and endogenous progesterone before supplementation and adherence.

Absence of a corpus luteum may also matter beyond implantation because the corpus luteum secretes vasoactive products in addition to progesterone. Observational studies and physiological work have linked programmed/no-corpus-luteum FET with altered maternal cardiovascular adaptation and higher risks of hypertensive disorders compared with ovulatory FET [73,74,75,76]. Confounding remains possible, but this signal is an important reason not to define cycle preparation solely by convenience or early pregnancy rate.

### 7.5. What Serum Progesterone Can—And Cannot—Tell Us

Serum progesterone is useful as an exposure and adherence marker, especially in programmed FET. A 2021 systematic review identified 21 cohort studies and meta-analyzed 16, representing 6175 cycles; 5881 were hormone-replacement FET and only 294 natural-cycle FET [15]. In the principal vaginal-progesterone/blastocyst comparisons using thresholds below 10 ng/mL, higher progesterone was associated with more ongoing pregnancy or live birth (RR 1.47, 95% CI 1.28–1.70) and less miscarriage (RR 0.62, 95% CI 0.50–0.77). These are observational associations, not a validated treatment rule. Across studies, cutoffs ranged from 5 to 53.2 ng/mL, dose-to-sampling intervals from 1 to 12 h and assay imprecision varied; only three studies were restricted to euploid embryos. The evidence therefore does not establish one universal cutoff, particularly at higher thresholds or across routes and cycle types (Table 5).

Four sources of non-equivalence must be considered. First, route changes the relationship between serum and uterine exposure. Second, progesterone secretion and absorption are time-dependent; a sample has meaning only relative to the last dose or endogenous pulse. Third, assay equivalence cannot be assumed. In 254 IVF patients sampled around the hCG trigger, one direct immunoassay produced a median progesterone concentration of 4.8 nmol/L versus 1.5 nmol/L by LC–MS, with an estimated intercept bias of 3.2 nmol/L; the authors considered the direct assay unsuitable for quantitative use below 5 nmol/L [77]. This study demonstrates platform- and concentration-specific bias at low late-follicular concentrations; it does not validate luteal FET thresholds, condemn all immunoassays or quantify metabolite interference for every platform. Fourth, serum exposure does not measure endometrial response or receptor competence. A value can be adequate while tissue response is impaired, or low while local vaginal delivery is substantial.

Thresholds should therefore be formulation-, route-, assay- and sampling-time-specific. Reporting only “progesterone on transfer day” is insufficient. Studies should record exact clock time, interval from the preceding dose, formulation, dose, route, adherence, embryo age and cycle type. Where feasible, a pharmacokinetic area-under-the-curve or repeated-measures approach is more informative than a single dichotomized value.

### 7.6. Rescue Supplementation: Promising Strategy, Unresolved Causal Effect

“Rescue” usually means adding or intensifying progesterone after a low serum result. A 2023 systematic review and meta-analysis of seven observational studies (5927 patients) found that outcomes in rescued low-progesterone cycles were similar to those in cycles with adequate concentrations, but graded the evidence as observational and unable to establish the untreated counterfactual [78]. Similarity after rescue can mean efficacy, selective treatment of patients with a favourable prognosis, threshold misclassification or residual confounding.

More recent cohorts and controlled studies have expanded the evidence base but not eliminated heterogeneity [79,80,81,82,83,84]. Studies use different low-value thresholds, sampling days, routes, rescue doses and outcomes. Rescue in a programmed cycle is also biologically different from supplementation in a true natural cycle. An ideal trial would randomize patients below a prespecified, assay-validated threshold to a protocolized rescue strategy or control, stratify by route and body size, and use live birth and maternal safety as outcomes. The threshold and analytic plan should be registered before outcomes are known.

Until then, serum monitoring and rescue may be reasonable components of a local quality-controlled protocol, particularly where a regimen has known absorption variability, but they should not be presented as universally validated precision medicine.

## 8. An Integrated Precision Framework

### 8.1. Four Linked Levels

**Figure 6:**
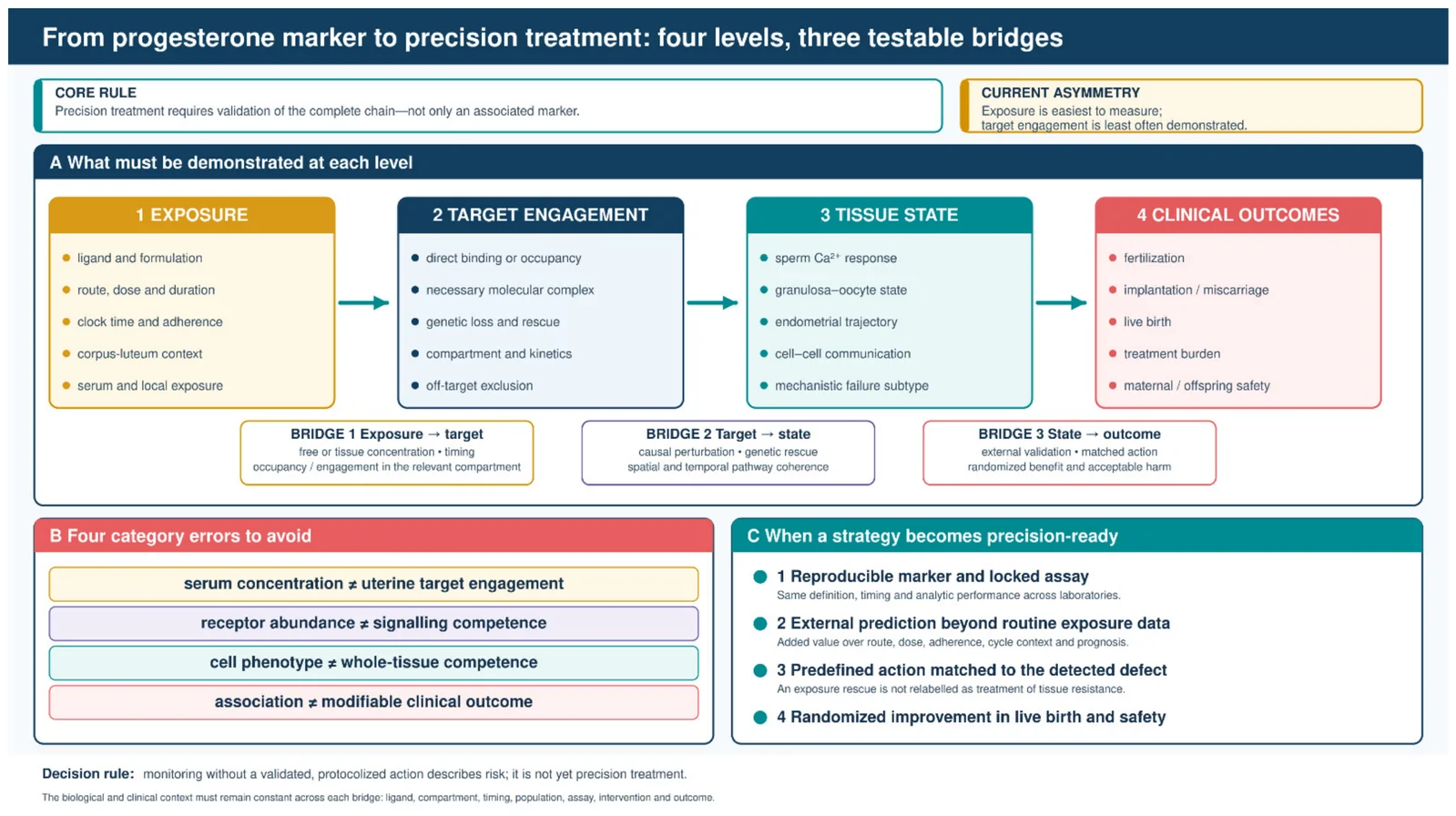
Exposure–target engagement–tissue state–clinical outcome framework for progesterone precision medicine. (**A**) The four levels are connected by three testable inferential bridges. Exposure must be linked to target engagement through tissue or free concentration, timing, occupancy, and engagement in the relevant compartment. Target engagement must be linked to tissue state through causal perturbation, genetic rescue, and spatial and temporal pathway coherence. Tissue state must be linked to outcome through external validation, a matched action, and evidence of clinical benefit with acceptable harm. (**B**) The framework prevents four category errors: serum concentration is not uterine target engagement; receptor abundance is not signalling competence; a cell phenotype is not whole-tissue competence; and association is not a modifiable clinical outcome. (**C**) A strategy becomes precision-ready only when the marker and assay are reproducible and locked, prediction is externally validated beyond routine exposure data, the action is predefined and matched to the detected defect, and a prospective randomized strategy improves live birth without unacceptable burden or safety consequences. Monitoring without a validated, protocolized action describes risk; it is not yet precision treatment.

The evidence across sperm, ovary, endometrium and luteal support can be organized into four linked levels (Figure 6).

1. **Exposure:** Ligand identity, formulation, route, dose, timing, adherence, local delivery, endogenous secretion and measured concentration.
2. **Target engagement:** Direct or functionally necessary interaction with a molecular target or complex in the relevant compartment.
3. **Tissue state:** The spatially and temporally integrated phenotype of sperm, follicular cells, oocyte or endometrium.
4. **Outcome:** Fertilization, blastocyst development, implantation, pregnancy loss, live birth and maternal or neonatal safety.

The framework prevents four common category errors. Serum concentration does not prove uterine target engagement. Receptor expression does not prove signalling competence. A molecular response in one cell type does not prove whole-tissue receptivity. An association with pregnancy does not establish that changing the measured marker will improve live birth.

In sperm, acute progesterone exposure produces a CatSper-dependent tissue-level response, while the proximal target remains unresolved. In the follicle, PGRMC perturbation changes cell and oocyte phenotypes, but ligand binding and human clinical relevance are less certain. In endometrium, classical PGR target engagement is well established, whereas the mapping from exposure to a coordinated receptive state varies among individuals. In programmed FET, route and adherence strongly influence exposure, but serum monitoring captures only one part of the pathway.

### 8.2. A Mechanism-Aware Clinical Decision Sequence

A pragmatic decision sequence begins with cycle context. In programmed FET, support is replacement and must be robust to missed or variably absorbed doses. In an ovulatory cycle, supplementation sits on top of endogenous secretion and must be timed relative to ovulation. Next, select a regimen with randomized or high-quality comparative evidence in that context. Patient preference, previous injection intolerance, vaginal factors, adherence risk, body size and local laboratory validation may then refine route and formulation.

If serum monitoring is used, the clinic should define the assay, sampling time relative to dosing, threshold and rescue action as one validated protocol. Ad hoc interpretation of a result using a threshold derived from another route or laboratory can create false precision. A low value should first trigger checks of timing, administration and adherence. A rescue dose addresses exposure failure; it is not a validated treatment for receptor, chromatin, metabolic or inflammatory resistance.

Repeated implantation failure requires a separate reasoning step. Before attributing failure to progesterone resistance, embryo competence, uterine anatomy, transfer factors and the accuracy of progesterone exposure should be reviewed. Molecular testing is most informative when it identifies a mechanistic subgroup and a matched intervention, not when it simply produces a receptivity label.

### 8.3. Safety Belongs Within, Not After, the Framework

Live birth cannot be the only downstream outcome. Route and cycle context can alter patient burden, local adverse effects, acceptability and possibly maternal vascular adaptation. Oral dydrogesterone and natural progesterone are not pharmacologically identical, so fetal and neonatal safety data should remain compound-specific. Current ART evidence is generally reassuring for commonly used regimens, but rare outcomes and long-term offspring health require registries with formulation, dose and timing recorded accurately.

The corpus-luteum signal illustrates why safety must be mechanistically interpreted. If ovulatory and programmed FET achieve similar live-birth rates, they may still differ in maternal vascular biology because progesterone replacement does not recreate all corpus-luteum products. This is a cycle-context effect, not evidence that progesterone itself causes or prevents hypertensive disease.

## 9. Experimental and Clinical Research Priorities

### 9.1. Resolve Target Identity with Orthogonal Methods

The field needs fewer receptor labels inferred from phenotype and more direct target-engagement studies (Table 6). For rapid sperm signalling, native-membrane chemoproteomics, photoaffinity probes and electrophysiology should be integrated. For PAQR proteins, binding and signalling should be demonstrated at endogenous abundance with genetic rescue. For PGRMC1/2, perturbations that separately disrupt haem binding, complex assembly, localization and candidate steroid interaction are needed. Ligand concentrations should be justified by free tissue exposure rather than nominal culture-media dose.

### 9.2. Preserve Space and Time in Tissue Models

Bulk endometrial or pooled follicular measurements erase the cell populations in which progesterone acts. Spatial transcriptomics, proteomics and metabolomics should be paired with histology and functional readouts. Longitudinal sampling is difficult in endometrium because biopsy perturbs the tissue; carefully validated organoids, assembloids and microphysiological systems can complement in vivo sampling. Models should include epithelial, stromal, immune and vascular components as appropriate and reproduce estrogen priming before progesterone exposure.

In ovarian research, each follicle should be treated as a linked system rather than averaging fluid and cells across follicles. Molecular data from mural granulosa, cumulus and fluid should be connected to the matched oocyte and embryo. Human validation should be planned before animal signatures are promoted as biomarkers.

### 9.3. Standardize Pharmacokinetic Reporting

Every clinical progesterone study should report formulation, manufacturer where relevant, nominal and actual dose, route, application time, adherence, blood-sampling clock time, interval since the preceding dose, assay platform, lower limit of quantification and cycle context. Serum values should not be converted between assays without a validated method comparison. Continuous analyses and non-linear models are preferable to post hoc threshold hunting.

For vaginal regimens, studies should explicitly address the dissociation between serum and local uterine delivery. A nested pharmacokinetic design with repeated serum measurements and, when ethically feasible, tissue or uterine-fluid measurements could identify whether low serum reflects global underexposure or preferential local delivery. Tissue sampling should not be assumed to be outcome-neutral.

### 9.4. Test Biomarker Strategies, Not Biomarkers in Isolation

A prognostic association is only the first step. The clinically relevant intervention is a strategy: measure a marker at a prespecified time; classify it with a locked algorithm; deliver a defined action; and improve a patient-important outcome. Trials of progesterone monitoring should therefore randomize the complete strategy or, among patients below a validated threshold, the rescue action. They should avoid excluding cancellations or early losses in ways that favour one arm.

Biomarker trials for endometrial resistance should require analytic validity, biological plausibility and a matched therapy. Subgroups and algorithms must be locked before external validation. The outcome hierarchy should include live birth, miscarriage, treatment burden, maternal complications and neonatal health—not implantation alone.

### 9.5. Design Route and Cycle Trials Around Non-Inferiority Correctly

Patient-friendly progesterone regimens are well suited to non-inferiority trials, but margins must be clinically justified and both intention-to-treat and per-protocol analyses should be reported. A non-significant superiority test does not establish equivalence. Cancellations, deviations, missed doses and rescue should remain visible in the assigned strategy. Trials comparing natural and programmed FET should include maternal cardiovascular and hypertensive outcomes and should be sufficiently large for uncommon harms.

### 9.6. Build Compound-Specific, Long-Term Safety Evidence

Registries should distinguish natural progesterone, dydrogesterone and other progestogens, as well as route and gestational exposure windows. Linkage to obstetric, neonatal and child-health data would allow assessment of rare congenital, neurodevelopmental and metabolic outcomes. Safety comparisons must address the strong confounding by infertility diagnosis, embryo testing, multiplicity, cycle protocol and maternal characteristics.

Table 6 converts these priorities into minimum study-design requirements. The central goal is not maximal data collection; it is a chain of evidence in which the exposure, molecular lesion, tissue consequence and treatment effect refer to the same biological context.

## 10. Conclusions

Progesterone in ART is not a single signal delivered to a single receptor and summarized by a single serum value. Classical PGR isoforms, PAQR-family proteins, PGRMC-associated complexes and the unresolved proximal sensor of sperm CatSper differ in molecular certainty and biological function. The strongest peripheral mechanistic claim is the rapid CatSper-dependent response of human sperm; the identity of its progesterone sensor is again open. Ovarian PGRMC1/2 biology is compelling but remains dominated by non-human and multifunctional-protein evidence. Endometrial competence is a temporally ordered, multicellular state in which classical PGR signalling intersects with transcriptional cofactors, metabolism, inflammation and tissue communication.

Clinical progesterone use should therefore be interpreted in its route and cycle context. Serum progesterone can identify some exposure failures, particularly in programmed FET, but it neither measures uterine concentration directly nor diagnoses tissue resistance. Rescue supplementation is biologically plausible and increasingly studied, yet universal thresholds and protocols are not established. Preserving the corpus luteum may also have consequences beyond progesterone replacement.

The exposure–target engagement–tissue state–outcome framework offers a disciplined path to precision treatment. It asks investigators to prove each inferential bridge and asks clinicians to validate the whole monitoring-and-action protocol rather than a number in isolation. Progress will depend on orthogonal target-engagement methods, compartment-resolved tissue models, standardized pharmacokinetics, properly designed biomarker-strategy trials and compound-specific long-term safety surveillance.

## Figures and Tables

**Table 1:** Progesterone-responsive molecular systems and the claim supported by current evidence.

System	Principal Location/Function in This Review	Strongest Supported Claim	What is Not Yet Established	Minimum Decisive Evidence
Classical PGR-A/PGR-B	Nucleus; chromatin; stromal and epithelial differentiation	Direct ligand-activated transcription with isoform- and context-specific programmes	That expression alone predicts tissue competence	Isoform-resolved perturbation, rescue, cistrome/transcriptome and phenotype
Membrane-associated classical PGR	Membrane-proximal rapid signalling	Classical PGR can participate in rapid signalling in selected systems	That every rapid progesterone response is mediated by membrane PGR	Compartment-specific loss/rescue plus rapid target engagement
PAQR5/7/8 (mPRγ/α/β)	Plasma membrane; second-messenger signalling	Progesterone-associated rapid responses occur in several expression and tissue models	Uniform endogenous direct binding and physiological necessity across reproductive tissues	Quantitative binding, endogenous genetic rescue and orthogonal signalling assays
PGRMC1/PGRMC2	ER/membrane complexes; haem biology; trafficking; survival and division	Perturbation alters ovarian and endometrial progesterone-associated phenotypes	Conventional receptor status or direct binding as the cause of every phenotype	Separation-of-function variants, complex mapping and target-engagement assays
CatSper pathway	Human sperm principal-piece membrane	Progesterone rapidly evokes CatSper-dependent Ca^2+^ influx	Identity of the proximal progesterone sensor	Native chemoproteomics combined with CatSper electrophysiology and genetic rescue
ABHD2	Sperm membrane/lipid regulation	May modulate lipid signalling in sperm	Requirement of hydrolase activity or a settled direct progesterone-sensor role	Catalytic-dead/null comparison, binding and rescue in human-relevant systems

**Table 2:** Peripheral progesterone biology in sperm and the ovarian follicle.

Compartment	Endpoint	Predominant Evidence	Translational Inference	Principal Limitation
Human sperm	Rapid Ca^2+^ influx and hyperactivated motility	Human electrophysiology and imaging; CatSper-deficient phenotypes	Candidate functional sperm assay	Proximal sensor unresolved; assay conditions vary
Granulosa/luteal cells	Survival/apoptosis; cell-cycle entry; mitosis/cytokinesis	Human granulosa/luteal-cell perturbation; rat cell models; mouse conditional deletion; bovine follicular and luteal models	PGRMC-associated regulation of survival and division; dependence is model- and context-specific	Species, culture and luteinization differences; multifunctional protein effects do not establish direct ligand binding
Oocyte	Meiotic arrest/progression and spindle organization	Mouse, bovine and zebrafish models	Potential link to competence	Species, developmental stage and stimulation context
Follicular fluid	Proteomic/metabolic environment	Human mass spectrometry and pathway analyses	Systems-level biomarker source	Association; variable pooling, timing and outcome linkage

**Table 3:** Endometrial response layers and representative failure modes.

Layer	Representative Nodes	Normal Function	Example Failure Mode	Measurement Caveat
Exposure	Route, dose, adherence, endogenous corpus luteum	Appropriate duration and local availability	Missed dose, variable absorption, mistimed start	Serum is route- and time-dependent
Receptor/chromatin	PGR-A/B; FOXO1, GATA2, IRF4; PGRMC1/2	Transcriptional reprogramming and organelle support	Isoform imbalance, methylation, cofactor deficiency	Bulk abundance misses activity, timing and cell type
Metabolism	IRS2, AMPK/SIRT1/PDK4	Energetic support of decidualization	Hyperandrogenic/metabolic impairment	In vitro decidualization may not reproduce whole tissue
Tissue communication	Epithelium, stroma, glands, immune cells	Receptivity, tolerance and controlled inflammation	Disrupted paracrine or prostaglandin signalling	Biopsy timing and composition alter signatures
Vascular state	Spiral vessels and vasoactive mediators	Implantation and maternal adaptation	No-corpus-luteum endocrine context	Strong potential for clinical confounding

**Table 4:** Clinically used progesterone/progestogen routes: advantages and interpretive limits.

Route/Compound	Exposure Characteristic	Potential Advantage	Principal Limitation	Interpretation of Serum Progesterone
Vaginal micronized progesterone/gel	High local uterine exposure in small PK studies; variable serum	No injection; potentially high local exposure	Leakage, irritation, adherence and absorption variability	Serum may understate local exposure; no universal tissue ratio
Intramuscular progesterone in oil	Sustained systemic exposure	Robust replacement evidence in programmed FET	Pain, inflammation, rare abscess; treatment burden	Reflects systemic exposure but remains time-dependent
Subcutaneous progesterone	Systemic exposure with smaller-volume injection	Patient acceptability and convenient dosing	Local reactions; fewer large comparative trials	Requires formulation- and sampling-specific validation
Oral dydrogesterone	Orally active progestogen; distinct metabolites	Convenience and phase III fresh-IVF evidence	Not natural progesterone; compound-specific safety and mechanisms	Standard progesterone assays do not measure dydrogesterone effect
Oral micronized progesterone	Marked first-pass metabolism	Convenient	Sedation and variable bioavailability; limited role as sole ART support	Parent progesterone may incompletely represent active metabolites/tissue effect

**Table 5:** Clinical interpretation of progesterone monitoring and rescue in FET.

Question	Best Current Answer	Certainty/Limitation	Practical Implication
Is low serum progesterone associated with poorer outcome?	Often yes in programmed FET using vaginal progesterone	Observational; mainly vaginal P plus blastocyst HRT-FET; thresholds and assays vary	Treat as a protocol-specific exposure signal
Is 10 ng/mL a universal cutoff?	No	Assay, route, timing, formulation and cycle context differ	Do not import a threshold from another protocol
Does serum concentration equal endometrial exposure?	No	Historical concept; small tissue PK studies; formulation- and patient-specific	Interpret alongside route and dosing time
Does rescue normalize outcomes?	Observational syntheses are compatible with benefit	Treated-only comparisons and confounding limit causality	Use a predefined local protocol; disclose uncertainty
Should all FET cycles be monitored?	Evidence is insufficient for a universal mandate	No definitive biomarker-strategy trial across regimens	Monitor when a validated threshold and action exist
Does a normal serum value exclude resistance?	No	Serum measures exposure, not tissue response	Investigate other causes before escalating dose repeatedly

**Table 6:** Research priorities and minimum design features.

Priority	Essential Design Features	Outcome That Would Change Practice
Identify the human sperm progesterone sensor	Native target engagement; CatSper electrophysiology; ABHD2 separation-of-function rescue	Reproducible causal sensor–channel pathway and diagnostic assay
Define PGRMC function in human follicles	Follicle-resolved multi-omics; matched cells/oocyte; genetic rescue	Human-validated mechanism predicting competence beyond routine variables
Classify endometrial response failure	Spatial cell-state map; exposure measured; external validation	Mechanistic subtype with a matched effective intervention
Validate serum monitoring	Locked assay/time/threshold/action; randomized strategy trial	Higher live birth without excess burden or harm
Test rescue	Randomization below a prespecified threshold; route stratification	Causal improvement in live birth and acceptable safety
Compare FET cycle strategies	Intention-to-treat including cancellation; obstetric and neonatal follow-up	Patient-centred selection incorporating effectiveness and maternal safety
Establish long-term safety	Compound-, route- and timing-specific registry linkage	Exclusion or quantification of rare and long-term offspring risks

## Data Availability

No new data were created or analyzed in this narrative review. The search strategies used to identify the literature are provided in the Supplementary Materials.

## Supplementary Materials

The following supporting information can be downloaded at: https://www.mdpi.com/article/10.3390/ijms27208944/s1.
