# Supplementary material for: Progesterone Beyond Pituitary Suppression in Assisted Reproduction: Peripheral Signalling, Gamete Biology, Endometrial Competence and Precision Therapeutic Use
Source: Int J Mol Sci. 2026 Oct 9;27(20):8944. doi: 10.3390/ijms27208944 (PMC13704441; doi:10.3390/ijms27208944)
Supplement: Supplementary file 1 [file ijms-27-08944-s001.zip › ijms-4565532-supplementary.pdf]

Supplementary Materials

Table S1. Reproducible PubMed/MEDLINE search concepts

Database: PubMed/MEDLINE. Final search date: 29 August 2026. Searches were supplemented by backward and forward citation tracing. Priority full texts available to the authors were inspected through 30 August 2026.

Concept	Query
Pgr Architecture	(progesterone receptor[Title/Abstract] OR PGR-A[Title/Abstract] OR PGR-B[Title/Abstract]) AND (isoform OR cistrome OR rapid OR nongenomic) AND (reproduction OR endometrium OR ovary)
Paqr Mpr	(membrane progestin receptor OR PAQR7 OR PAQR8 OR PAQR5) AND progesterone
Pgrmc	(PGRMC1 OR PGRMC2) AND (progesterone OR ovary OR oocyte OR granulosa OR endometrium)
Catsper	(progesterone AND CatSper AND human sperm) OR (ABHD2 AND sperm)
Ovary Oocyte	progesterone AND (PGRMC1 OR PGRMC2) AND (granulosa OR oocyte OR follicle) AND (survival OR mitosis OR meiosis OR competence)
Endometrium Network	progesterone receptor AND (decidualization OR endometrial receptivity) AND (FOXO1 OR GATA2 OR cistrome OR stromal OR epithelial)
Resistance	progesterone resistance AND (endometriosis OR PCOS OR endometrium) AND (mechanism OR molecular)
Uterine Pk	progesterone AND (uterine first pass OR endometrial tissue concentration OR vaginal pharmacokinetics)
Fresh Lps	luteal phase support AND (fresh embryo transfer OR IVF) AND (randomized OR meta-analysis OR network meta-analysis)
Fet Cycles	frozen embryo transfer AND (natural cycle OR programmed cycle OR artificial cycle) AND (randomized OR meta-analysis OR live birth)
Corpus Luteum	frozen embryo transfer AND corpus luteum AND (hypertensive OR cardiovascular OR preeclampsia OR obstetric)
Serum Progesterone	frozen embryo transfer AND serum progesterone AND (threshold OR assay OR timing OR live birth)
Rescue	frozen embryo transfer AND low serum progesterone AND rescue AND (live birth OR pregnancy)
Dydrogesterone	dydrogesterone AND luteal phase support AND (randomized OR meta-analysis OR pharmacology)
Author Group	(Lukaszuk K[Author] OR Woclawek-Potocka I[Author] OR Jakiel G[Author] OR Lukaszuk J[Author]) AND (endometrium OR follicular fluid OR oocyte OR progesterone OR prostaglandin)

Table S2. Citation and access audit

No.	PMID	Year	DOI	Full-text status
1	38110672	2024	10.1038/s41574-023-00921-5	Priority full text pending
2	26148507	2015	10.1002/14651858.CD009154.pub3	PMC open
3	29025055	2017	10.1093/humrep/dex285	Library/publisher
4	36384863	2022	10.1016/j.tem.2022.10.001	Library/publisher
5	25406186	2015	10.1093/humupd/dmu056	PMC open
6	26203174	2015	10.1095/biolreprod.115.131508	Library/publisher
7	36463977	2023	10.1016/j.pharmthera.2022.108326	PMC open
8	21412339	2011	10.1038/nature09767	Full text verified 30 August 2026
9	21412338	2011	10.1038/nature09769	Full text verified 30 August 2026
10	26989199	2016	10.1126/science.aad6887	PMC open

No.	PMID	Year	DOI	Full-text status
11	42212558	2026	10.1093/humrep/deag085	PMC open
12	19417032	2009	10.1210/jc.2009-0147	PMC open
13	25253729	2014	10.1095/biolreprod.114.122986	PMC open
14	30877763	2019	10.1093/biolre/ioz043	PMC open
15	34384594	2021	10.1016/j.fertnstert.2021.07.002	Full text verified 30 August 2026
16	25781565	2015	10.1210/en.2014-1566	PMC open
17	25875046	2015	10.1210/me.2014-1363	PMC open
18	34217103	2021	10.1016/j.repbio.2021.100529	Library/publisher
19	24065879	2013	10.3389/fnins.2013.00168	PMC open
20	35595324	2022	10.1210/endocr/bqac078	PMC open
21	30595525	2019	10.1016/j.rbmo.2018.11.017	Library/publisher
22	32432564	2020	10.1515/hmbci-2019-0067	Library/publisher
23	37547474	2023	10.3389/fcell.2023.1221578	PMC open
24	22354039	2012	10.1038/emboj.2012.30	PMC open
25	28507119	2017	10.1073/pnas.1700367114	PMC open
26	23530196	2013	10.1073/pnas.1216588110	PMC open
27	26453676	2015	10.1093/humrep/dev243	PMC open
28	16513825	2006	10.1210/en.2006-0114	Priority full text pending
29	15329328	2004	10.1095/biolreprod.104.028076	Full text verified 30 August 2026
30	27260975	2016	10.1080/15384101.2016.1192731	PMC open
31	27848973	2016	10.1038/srep36869	PMC open
32	37567656	2023	10.1016/j.animal.2023.100783	Library/publisher
33	30319543	2018	10.3389/fendo.2018.00560	PMC open
34	30306772	2018	10.1530/REP-18-0155	PMC open
35	28005395	2017	10.1210/en.2016-1701	PMC open
36	28658951	2017	10.1021/acs.jproteome.7b00366	Library/publisher
37	29530678	2019	10.1016/j.jprot.2018.03.010	Library/publisher
38	39126000	2024	10.3390/ijms25158431	PMC open
39	39519300	2024	10.3390/ijms252111749	PMC open
40	32393337	2020	10.1186/s12958-020-00598-9	PMC open
41	25584414	2015	10.1210/me.2014-1292	PMC open
42	32335672	2020	10.1210/endocr/bqaa070	PMC open
43	31748790	2020	10.1210/endocr/bqz021	PMC open
44	36821428	2023	10.1093/molehr/gaad007	PMC open
45	36135172	2022	10.3390/cimb44090260	PMC open
46	35526781	2022	10.1016/j.steroids.2022.109040	Library/publisher
47	38308254	2024	10.1186/s12958-024-01188-9	PMC open
48	38452349	2024	10.1093/humrep/deae044	Full text verified 30 August 2026
49	22085558	2012	10.1016/j.mce.2011.10.035	Library/publisher
50	20104427	2010	10.1055/s-0029-1242991	PMC open
51	28423456	2017	10.1111/aogs.13156	Library/publisher
52	31387263	2019	10.3390/ijms20153822	PMC open
53	34665857	2022	10.1210/clinem/dgab753	PMC open
54	24405633	2014	10.1186/1423-0127-21-2	PMC open
55	39080028	2024	10.1007/s00018-024-05362-5	PMC open
56	38011671	2024	10.1093/biolre/ioad161	Priority full text pending

No.	PMID	Year	DOI	Full-text status
57	9329850	1997	10.1111/j.1749-6632.1997.tb48550.x	Full text verified 30 August 2026
58	9194669	1997	10.1093/humrep/12.5.1073	Priority full text pending
59	8062942	1994	10.1016/s0015-0282(16)56935-0	Full text verified 30 August 2026
60	10928427	2000	10.1093/humrep/15.suppl_1.159	Library/publisher
61	31032676	2019	10.1080/09513590.2019.1603288	Library/publisher
62	38914570	2024	10.1038/s41598-024-64804-z	PMC open
63	28333318	2017	10.1093/humrep/dex023	PMC open
64	30304457	2018	10.1093/humrep/dey306	PMC open
65	33147288	2020	10.1371/journal.pone.0241044	PMC open
66	33992421	2021	10.1016/j.fertnstert.2021.04.013	Full text verified 30 August 2026
67	39223536	2024	10.1186/s12905-024-03337-z	PMC open
68	38944045	2024	10.1016/S0140-6736(24)00756-6	Full text verified 30 August 2026
69	20579989	2011	10.1016/j.fertnstert.2010.05.019	Library/publisher
70	33307906	2021	10.1080/09513590.2020.1854717	Library/publisher
71	35972453	2022	10.1093/humrep/deac181	PMC open
72	36574915	2023	10.1016/j.fertnstert.2022.12.035	Library/publisher
73	33748839	2021	10.1093/humupd/dmab003	PMC open
74	34437863	2022	10.1016/j.ajog.2021.08.018	Library/publisher
75	34568385	2021	10.3389/fmed.2021.727753	PMC open
76	38536596	2024	10.1007/s10815-024-03042-8	PMC open
77	33626843	2016	10.1373/jalm.2016.020123	Full text verified 30 August 2026
78	36781098	2023	10.1016/j.fertnstert.2023.02.007	Library/publisher
79	39527238	2025	10.1007/s10815-024-03309-0	PMC open
80	40757044	2025	10.18502/jri.v26i1.18779	PMC open
81	41098624	2025	10.3389/frph.2025.1547939	PMC open
82	41115974	2025	10.1007/s10815-025-03690-4	PMC open
83	41622134	2026	10.1186/s12884-026-08645-w	PMC open
84	42087457	2026	10.1002/ijgo.71059	Library/publisher
